# Literary Notices

**Published:** 1872-10

**Authors:** 


### LITERARY NOTICES.

*Scribner's Monthly:* The New York Evening Post says of Arthur Bonnicastle, Dr. Holland's Serial, commenced in Scribner's Monthly for November:

"Only one chapter of twelve pages is given, but this is enough to awaken interest. There is a good deal of quiet humor in the dialogue, and the characters are fresh and natural, while the descriptive passages, it need scarcely be said, is fluent and fascinating. Much of the effect of Dr. Holland's writing is to be attributed to the ease and grace of the composition. The dramatic promise of Arthur Bonnicastle, however, is such as to make us regret that the author has not been writing novels these many years."

*Words and Their Uses. Past and Present.* A Study of the English Language. By Richard Grant White. New York: Sheldon & Co. 677 Broadway. 1872.

No more readable work on the uses of words and the study of the English language has been issued from the press, than the one before us. It is a book all can study with profit, and one parents should place in the hands of their children.

*Schools and School-Masters.* From the Writings of Charles Dickens. Edited by T. J. Chapman, M. A., A. S. Barnes & Co., New York and Chicago.

This is a beautiful volume, containing some of the finest passages drawn from the works of Dickens. It contains the following sketches: Dotheboys Hall; The School at Dr. Blimber's; The School at Salem House; The School at Dr. Strong's. It is an amusing and attractive volume, which the young would take special delight in reading.

*Recollections of Past Life.* By Sir Henry Holland, M. D., F. R. S., D. C. L., etc., etc., President of the Royal Institution of Great Britain, etc. New York: D. Appleton & Co. 1872.

Dr. Holland has written a most charming autobiography. His life covers some of the most stirring events of modern history, stretching over eighty years. An active practitioner, and eminently successful in every respect, he was also a great traveller, visiting almost every portion of the globe.

*Secret History of the French Court Under Richelieu and Mazarin;* Or, Life and Times of Madame de Chevreuse. By Victor Cousin, Author of "The True, the Beautiful and the Good, etc., etc. Translated by Mary L Booth. New York: James Miller, Publisber, 647 Broadway. 1872.

This is a charming history of the times of Richelieu, written in the beautiful, flowing style of Cousin. The secret history of the French Court at the time of Richelieu, as told by Cousin, has the charm and drapery of romance more than actual history, and which, in the volume before us, cannot fail to both interest and instruct the reader.

*Bibliographical.* Ovarian Tumors: Their Pathology, Diagnosis and Treatment, especially by Ovariatomy. By E. Randolph Peaslee, M.D., L.L.D., Professor of Gynæcology in the Medical Department of Dartmouth College; Attending Surgeon of the New York Woman's Hospital, etc., ect., with fifty-six illustration. New York: D. Appleton & Co., 549 and 551 Broadway. 1872.

Professor Peaslee stands among the first Gynæcologists of this, or any other county; and his work, exhaustive and accurate, on Ovarian Tumors, while it will add to his well-earned reputation, affords the profession a work much needed.

No work, prior to this, has covered the entire ground of the subjects treated, and the distinguished author enriches its pages, not only by his thorough knowledge of his subjects, but by a pratical and devoted study of them for the last twenty-five years. Professor Peaslee does not fetter his convictions by a blind obedience to the opinions of others, and truly remarks, that "the time for oracular utterances in our art has passed; and every magisterial assertion should be challenged." Hence he can truthfully claim that "he has drawn from his own experience whatever afforded (and richly, too,) a better illustration of a special point than he could obtain from another source."

"The first part of the work includes the normal anatomy of the ovary, and the pathological anatomy, the pathology, diagnosis and treatment of Ovarian tumors, excepting by ovariotomy. The second part is devoted to ovariotomy alone, including its history, statistics, practical details, and after treatment. It aims to decide all practical questions by the aggregate experience of all ovariotomists up to the present time."

It is almost superfluous to say that the work is one the profession should prize—one that every earnest practitioner should possess. The work is beautifully printed, and handsomely illustrated.

*The Physiology of Man;* Designed to represent the existing state of Physiological Science, as applied to the functions of the Human Body. By Austin Flint, Jr., M.D., Professor of Physiology and Physiological Anatomy in the Bellevue Hospital, etc. Nervous System. New York: D. Appleton & Co.

The volume before us, consisting of 461 pages, and forming one of five series, is devoted exclusively to the Nervous System. The Publishers, with commendable zeal, were "desirous of presenting a complete work on the Pysiology and Pathology of the Nervous System." Professors Flint and Hammond heartily concurred in the plan. Professor Hammond's has already been noticed.—which, together with the work of Professor Flint, forms the most thorough and exhaustive works upon the Physiology, Pathology and treatment of Nervous Diseases.

The work of Professor Flint should, therefore, be studied by all who have, or expect to, read that of Professor Hammond.

The plan and scope of the volume before us may be seen by reference to its contents:

Chapter i, treats of the Physiological divisions and structure of the Nervous System. Chapter ii, Motor and Sensory Nerves. Chapter iii, General properties of the nerves. Chapter iv, Spinal nerves—motor nerves of the eye-ball. Chapter v, Motor nerves of the face. Chapter vi, Spinal accessory and sublingual nerves. Chapter vii, Trifacial, or trigeminal nerves. Chapter viii, Pneumogastric, or par vagum nerves. Chapter ix, Physiological anatomy and general properties of the spinal cord. Chapter x, Action of the spinal cord as a conductor. Chapter xi, Action of the spinal cord as a nerve-centre. Chapter xii, The cerebral hemispheres. Chapter xiii, The cerebellum. Chapter xiv, Ganglia at the base of the encephalon. Chapter xv, Sympathetic nervous system. Chapter xvi, Sleep.

The Physiological series of Professor Flint, when completed, will be the most accurate and thorough ever presented to the English reader, an honor alike to this country and the profession he represents. That they will be prized and read by the profession of both continents we confidently predict.

*Hysterology.* A Theatise Discriptive and Clinical, on the Diseases and the Displacements of the uterus. By Edwin Nesbit, Chapman M. A. M. D., late Professor of Obstetrics; Diseases of Women and Children, and Clinical Midwifery in the Long Island College Hospital. New York: William Wood & Co., 27 Great Jones Street. 1872.

The author says: "My purpose, an humble one, is simply the delineation, from personal observation, of the histories, symptoms, pathology and treatment of the special disorders of the nongravid uterus. To effect this I shall attempt the difficult task of drawing my descriptions from the open page of Nature."

The author's pathology excludes inflammation as the usual factor in the production of uterine disorders, which he regards as results of congestion, similar to that produced by menstruation and pregnancy, "but of a continuous character."

His treatment, for the most part, is simple—of local applications of solutions of nitrate of silver and direct abstractions of blood, by scarifications and leeches to the neck of the uterus. His treatment in the main, is based upon the idea that after every menstruation there is an involution of the substance of the uterus as the result of the physiological congestion consequent upon that function and similar to that resulting from pregnancy. Hence in order to hasten and assist this involution, he regards as the one essential element in the successful treatment of uterine enlargement, ulcerations, hypertrophy, displacements, etc.

The author discards the pathology and the treatment based upon it of the prominent gynæcologists of the day. In this many of the profession will not agree with him. The work, however, should be read; serving, as it will, a good purpose in spite of the new pathology upon which the treatment is founded.

It is handsomely printed, reflecting great credit upon the publishers, Messrs. Wood & Co.

*Undine; Or, The Water-Spirit* Also, Sintram and His Companions. From the German of Freidrich De La Motte Fouque. New York: James Miller, 647 Broadway.

This is a beautifully bound volume, and contains two of the most attractive stories ever written. It would be a rare treat to the young to secure this volume, and parents could confer no greater pleasure on themselves than to make them happy by a gift of "Undine" and "Sintram."

Page(s) missing

